# Diagnostic utility of multidetector CT scan in penetrating diaphragmatic injuries: A systematic review and meta-analysis

**DOI:** 10.1007/s10140-023-02174-1

**Published:** 2023-10-04

**Authors:** Amir Hassankhani, Melika Amoukhteh, Parya Valizadeh, Payam Jannatdoust, Liesl S. Eibschutz, Lee A. Myers, Ali Gholamrezanezhad

**Affiliations:** 1 Department of Radiology, Keck School of Medicine, University of Southern California (USC), 1441 Eastlake Ave Ste 2315, Los Angeles, CA 90089 USA; 2 Department of Radiology, Mayo Clinic, Rochester, MN USA; 3 School of Medicine, Tehran University of Medical Sciences, Tehran, Iran; 4 Department of Diagnostic and Interventional Imaging, McGovern Medical School, University of Texas Health Science Center at Houston, Houston, TX USA

**Keywords:** Computed tomography, Diagnosis, Diaphragmatic injuries

## Abstract

Penetrating diaphragmatic injuries pose diagnostic and management challenges. Computed tomography (CT) scans are valuable for stable patients, but concern exists for missed injuries and complications in nonoperatively managed cases. The objective of this study was to explore the diagnostic utility of multidetector CT scan (MDCT) in identifying diaphragmatic injuries resulting from penetrating trauma. A systematic review and meta-analysis were conducted, following established guidelines, by searching PubMed, Scopus, Web of Science, and Embase databases up to July 6, 2023. Eligible studies reporting MDCT's diagnostic accuracy in detecting penetrating diaphragmatic injuries were included. Relevant data elements were extracted and analyzed using STATA software. The study included 9 articles comprising 294 patients with confirmed penetrating diaphragmatic injuries through surgical procedures. MDCT's diagnostic performance revealed a pooled sensitivity of 74% (95% CI: 56%-87%) and a pooled specificity of 92% (95% CI: 79%-97%) (Fig. two), with significant heterogeneity in both sensitivity and specificity across the studies. The Fagan plot demonstrated that higher pre-test probabilities correlated with higher positive post-test probabilities for penetrating diaphragmatic injury diagnosis using MDCT, but even with negative results, there remained a small chance of having the injury, especially in cases with higher pre-test probabilities. This study highlights MDCT's effectiveness in detecting diaphragmatic injury from penetrating trauma, with moderate to high diagnostic accuracy. However, larger sample sizes, multicenter collaborations, and prospective designs are needed to address observed heterogeneity, enhancing understanding and consistency in MDCT's diagnostic capabilities in this context.

**Supplementary Information:**

The online version contains supplementary material available at 10.1007/s10140-023-02174-1.

## Introduction

Accurate diagnosis and appropriate management of penetrating trauma to the diaphragm present a significant challenge [1, 2]. Thoracoabdominal penetrating traumas often result in diaphragmatic injuries, occurring in approximately 30% of cases [1]. However, these injuries can be difficult to detect due to nonspecific signs and symptoms, and around 7% of cases may be occult when occurring in isolation [1, 3]. Additionally, the small size of these injuries further complicates diagnosis, as minor tears can enlarge over time due to negative intrathoracic pressures, potentially leading to herniation of abdominal contents and subsequent complications [4–6].

The approach to thoracoabdominal trauma caused by penetrating injuries can be complex. Immediate operative exploration is necessary for hemodynamically unstable patients [2, 5] while patients with normal vital signs and no clear indications for surgery can be managed using various algorithms [2, 7, 8].

Despite the high sensitivity of operative exploration in detecting diaphragmatic injuries [2, 5, 6], this approach can still yield procedural complications in a significant number of cases [7, 9]. Therefore, a non-invasive approach to reliably detect diaphragmatic injuries in hemodynamically stable patients would help reduce unnecessary operations, minimize associated morbidity, and decrease costs [6, 9].

In the absence of clinical indications for surgery, stable patients with penetrating thoracoabdominal trauma are commonly evaluated using computed tomography (CT) scanning, particularly multidetector CT (MDCT) [2, 7, 11]. MDCT's high-resolution images in different planes have improved the detection accuracy of diaphragm injuries [2, 6, 8]. However, there remains a concern about missed diaphragmatic injuries in nonoperatively managed patients, as these injuries can go unnoticed and lead to life-threatening complications [7].

Considering these challenges and the potential benefits of MDCT in detecting penetrating diaphragm injuries, as supported by existing literature, this study aims to explore the diagnostic utility of MDCT in identifying diaphragmatic injuries resulting from penetrating trauma.

## Methods

In accordance with the Preferred reporting items for systematic reviews and meta-analyses (PRISMA) guidelines [12], a comprehensive literature search was conducted on July 6, 2023, across PubMed, Scopus, Web of Science, and Embase databases. Customized search terms were used for each database, including ("CT scan" OR "CT-scan" OR "computed tomography" OR "computerized tomography" OR "tomography, x-ray computed") AND ("diaphragm*") AND ("penetration" OR "penetrating trauma" OR "penetrating injur*" OR "penetrating wound*" OR "gunshot wound*" OR "stab wound*" OR "penetrating abdominal trauma" OR "penetrating abdominal injur*" OR "penetrating chest trauma" OR "penetrating chest injur*" OR "penetrating thoracic trauma" OR "penetrating thoracic injur*" OR "penetrating thoracoabdominal trauma" OR "penetrating thoracoabdominal injur*"). Additionally, a manual search of references from included studies was performed to ensure thorough coverage. The AutoLit platform, developed by Nested Knowledge in St. Paul, Minnesota, USA, was utilized for deduplication, screening, and data extraction purposes.

All studies reporting at least one of the diagnostic accuracy measures of MDCT in detecting diaphragmatic injuries resulting from penetrating trauma, including accuracy, sensitivity, specificity, positive predictive value (PPV), and negative predictive value (NPV), were included. There were no restrictions regarding the date, country of origin, patient characteristics, reference standard type utilized, or study design. Exclusions encompassed duplicate papers, non-English literature, case series with fewer than five eligible patients, case reports, conference abstracts, editorial comments, author responses, review articles, nonhuman studies, and irrelevant papers pertaining to the topic of interest.

The screening process involved an evaluation of the title, abstract, and/or full text of each study. Two authors independently assessed the articles, and any uncertainties or ambiguities were resolved through consultation with a senior coauthor. The extracted data from each eligible paper included the first author's name, publication year, country of origin, study design, sample size and characteristics, characteristics of diaphragmatic and associated injuries, CT device specifications, utilization of contrast material, reference standard method, and diagnostic accuracy measures of MDCT.

The quality of the included studies was assessed using the Quality Assessment of Diagnostic Accuracy Studies (QUADAS-2) tool [13]. The QUADAS-2 tool evaluates the risk of bias and applicability concerns in four primary domains: patient selection, index test, reference standard, and flow and timing. Each domain was independently evaluated based on specific criteria outlined in the tool, such as representativeness of the study population, blinding of test results, and completeness of outcome data. Ratings of "low," "high," or "unclear" were assigned to each domain assessment.

### Statistical analysis

The analysis involved calculating true positive, true negative, false positive, and false negative values, which were used to construct 2 × 2 tables presenting sensitivity and specificity data. To address cells in the 2 × 2 tables with zero values, a continuity correction was applied by adding 0.5 to each cell value. Summary effect estimates were obtained using a bivariate random effects model [14], allowing the creation of a summary receiver operating characteristic (SROC) curve and calculation of the area under the curve (AUC). Heterogeneity among studies was assessed using the I^2^ test [15], and subgroup analyses were conducted for I^2^ values exceeding 50% to explore potential sources of heterogeneity. Fagan plot analyses were performed, assuming pre-test probabilities of 25%, 50%, and 75%, to determine post-test probabilities for positive and negative results. Publication bias was evaluated using Deek's test [16], and if bias was present, the trim-and-fill method proposed by Duvall and Tweedie was used to create a symmetrical funnel plot and calculate an estimated summary value [17]. The statistical analysis was performed using the MIDAS user-made module for diagnostic test accuracy (DTA) meta-analysis [18] and STATA software (Version 17.0, Stata Corp, College Station, TX). A p-value less than 0.05 was considered statistically significant.

## Results

### Article screening and selection process

**Fig. 1:**
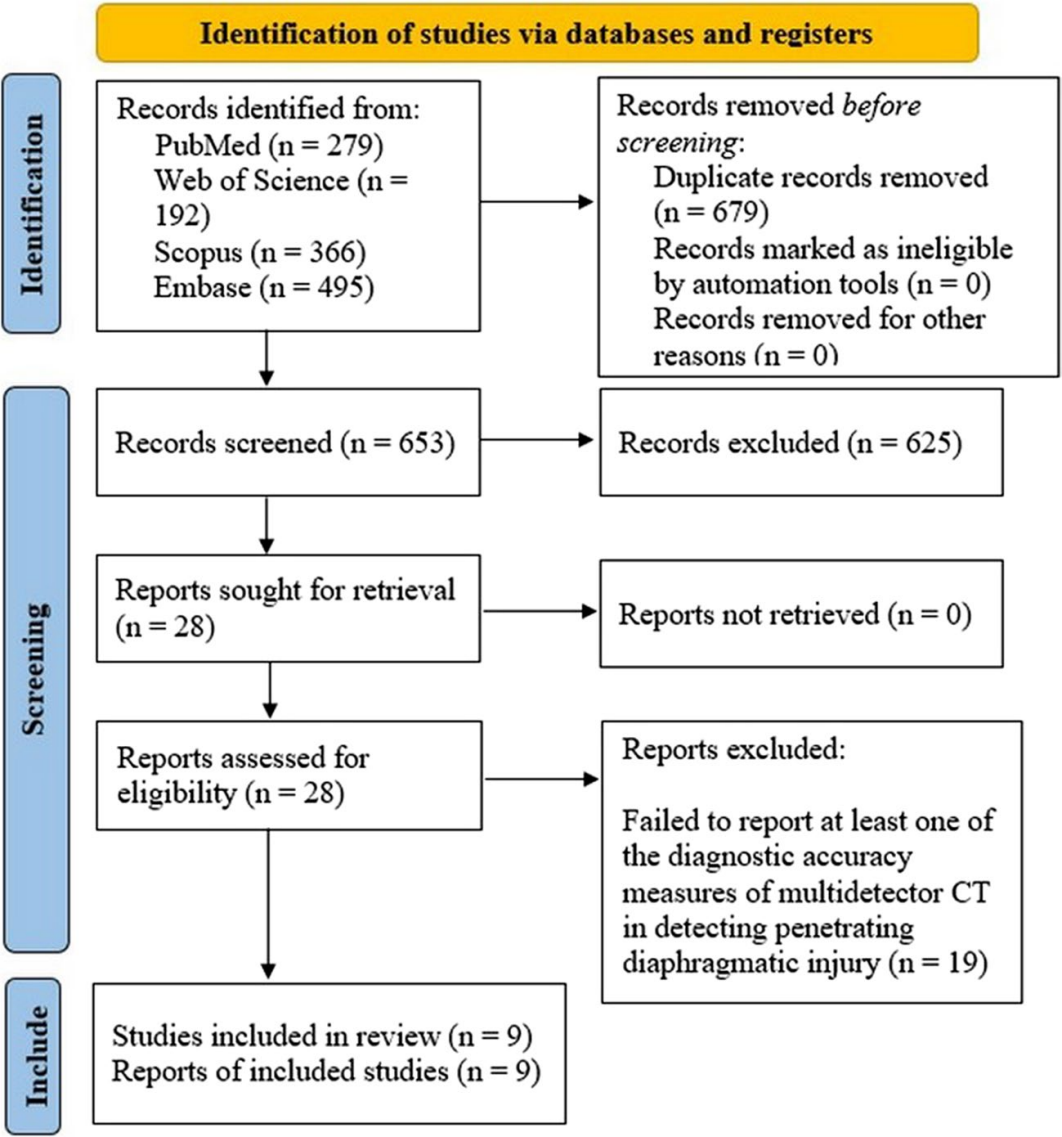
PRISMA flow diagram showing the review process. PRISMA: Preferred Reporting Items for Systematic Reviews and Meta-Analyses

Using a predefined search strategy, 1332 articles were initially identified. After removing duplicates, 653 papers were screened based on title and abstract, resulting in the exclusion of 625 articles. The full text of the remaining 28 papers was thoroughly reviewed, leading to the exclusion of 19 articles not aligned with the study's aim. Ultimately, 9 articles meeting the inclusion criteria were identified. A flow diagram following PRISMA guidelines (Fig. 1) summarizes the screening process and eligibility criteria.

### Characteristics of the included studies

The selected studies included in our analysis comprised of 7 cohort studies [2, 5–8, 19, 20] and 2 case–control studies [1, 21]. These studies were conducted in various countries, including the United States (n = 4), France, Colombia, Canada, Brazil, and Turkey (each n = 1). The age range of patients across the studies varied from 13 to 86 years, with the majority of patients being male.

Among the nine articles selected, the majority of studies utilized thoracic, abdominal, or a combination of both types of MDCT scans. Three studies used oral contrast agents [2, 8, 20], three studies employed rectal administration of contrast material [2, 7, 8], and seven studies utilized IV contrast agents [1, 2, 7, 8, 19–21]. It is important to note that some studies incorporated multiple routes of contrast administration. Table 1 provides a summary of the general characteristics of the examined studies.

**Table 1:** Characteristics of the included studies and patients

First author, year of publication	Country	Study design	Age (years)	Male (%)	CT type	CT device/Slice thickness	Contrast material
Augustin, 2019	France	Prospective cohort	NS	NS	Thoracoabdominal	A 64-row MDCT scanner/0.625 mm	NS (IV)
Bodanapally, 2009	United States	Retrospective cohort	29.6, (13–86) Mean, (Range)	86	Thoracic, Thoracoabdominopelvic, Abdominopelvic	MX 8000 or Brilliance 16 Power; Philips Medical Systems, Cleveland, OH (a 4- or 16-slice CT system)/3–5 mm	2% sodium diatrizoate (Hypaque sodium; Nycomed, Princeton, NJ); 600 ml IV and 1–1.5 L enema
Dreizin, 2013	United States	Case–control	32.6, (16–85) Mean, (Range)	92.6	Thoracoabdominal	Somatom; Siemens, Malvern, Pa (64-section MDCT)/1.5 mm	103 mL of IV ioversol, Optiray; Mallinckrodt, Hazelwood, Mo
Daza-Cajas, 2021	Colombia	Prospective cohort	23.2, (18–37) Mean, (Range)	90.75	Thoracic, Abdominal, Thoracoabdominal	GE LightSpeed; GE Healthcare, Milwaukee, WI, USA; 64 rows of detectors/ < 3 mm	NS (Non-contrast, IV or oral)
Leung, 2015	Canada	Case–control	NS	NS	Abdominal	a 64-slice MDCT scanner/3 mm	125 mL IV Omnipaque 300, GE Healthcare
Melo, 2011	Brazil	Prospective cohort	24.3, (6.8), (14–44) Mean, (SD), (Range)	96.77	Thoracoabdominal	GE LightSpeed; GE Healthcare, Milwaukee, WI, USA; 8 rows of detectors/1.25 mm	100 mL IV iobitridol, Henetix 300, Guerbet, Rio de Janeiro, Brazil; 400 mL oral iodinated contrast solution; 200 mL of the same solution rectally
Stein, 2007	United States	Retrospective cohort	29.6, (11.4), (13–86) Mean, (SD), (Range)	87.7	Thoracic, Abdominal, Thoracoabdominal	MX 8000 or Brilliance 16 Power; Philips Medical Systems, Cleveland, OH (a 4- or 16-slice MDCT scanner)/3 mm	Oral, rectal and 150 mL of I2 mg/mL of IV contrast material
Uhlich, 2018	United States	Retrospective cohort	NS	NS	NS	Koninklijke Philips NV, Amsterdam; a 256-slice CT scanner, and Philips Brilliance Power; a 64-slice machine/NS	NS
Yucel, 2015	Turkey	Prospective cohort	30, (15–61) Mean, (Range)	91	Thoracoabdominal	Multislice CT scanner	NS

*GE* General Electric, *IV* Intravenous, *MDCT* Multidetector computed tomography, *NS* Not specified

### Diaphragmatic injuries and CT findings

A total of 933 patients with penetrating trauma underwent both CT scan and the reference standard, resulting in the identification of 294 patients with penetrating diaphragmatic injury. Table 2 provides an overview of the general characteristics of diaphragmatic injuries resulting from penetrating trauma across all the included studies. Among these studies, bilateral injury was reported in only two studies [1, 20], while left-sided injury was the most commonly observed. Surgical exploration served as the reference standard in all of the studies included.

**Table 2:** Included patients, characteristics of penetrating diaphragmatic injury, and reference standard utilized in each study

First author, year of publication	Patients received both CT scan and reference standard (N)	Patients with PDI, confirmed by reference standard (N)	Right-sided injury (%)	Left-sided injury (%)	Bilateral injury (%)	Reference standard
Augustin, 2019	33	14	15.4	84.6	NS	Surgical exploration
Bodanapally, 2009	136	47	NS	mostly left-sided	NS	Surgical exploration
Dreizin, 2013	27	15	40.8	37	22.2	Surgical exploration
Daza-Cajas, 2021	119	36	22.9	55.5	17.6	Surgical exploration
Leung, 2015	17	9	NS	NS	NS	Surgical exploration
Melo, 2011	31	8	NS	NS	NS	Surgical exploration
Stein, 2007	154	50	NS	NS	NS	Surgical exploration
Uhlich, 2018	373	104	NS	NS	NS	Surgical exploration
Yucel, 2015	43	11	0	100	0	Surgical exploration

*NS* Not specified, *N* number, *PDI* Penetrating diaphragmatic injury

Regarding the mechanism of injury, two studies reported stab wounds [5, 19], while two studies specifically focused on gunshot injuries [8, 20]. Four studies documented cases involving both stab and gunshot wounds [1, 2, 7, 21], and one study did not provide specific information on the penetrating trauma mechanism [6]. Table 3 presents the diagnostic accuracy of MDCT scan in cases of diaphragmatic injury caused by penetrating trauma in the included studies.

**Table 3:** Summary of findings in each study

First author, year of publication	MOI	Important CT findings	Associated injuries	CT overall diagnostic accuracy
Augustin, 2019	SW	NS	Thoracic new findings (pneumomediastinum, hemopneumothorax, pneumothorax, hemothorax, hemopericardium, ribs/scapula fracture)	Sen:50; Spe: 95; PPV:88; NPV: 72
Bodanapally, 2009	SW (43.38%) GSW (46.62%) Steel reinforcing bar injury (0.73%)	Contiguous injury sign on both sides of the diaphragm (the most accurate); Herniation of abdominal viscera into the thorax and the Collar sign (the most specific); Dependent viscera sign; Discontinuous diaphragm; Diaphragmatic thickening sign (the least specific)	Hemothorax, hemoperitoneum, splenic injury	Sen:87.2; Spe: 72.4; Accuracy: 77
Dreizin, 2013	SW (40.74%) GSW (59.26%)	Contiguous injury sign on both sides of the diaphragm (the most sensitive); Transdiaphragmatic trajectory (highly specific); Diaphragmatic discontinuity	NS	Sen: 86.67; Spe: 75; PPV: 81.25; NPV: 81.82
Daza-Cajas, 2021	GSW (85.71%)	Contiguous injury sign on both sides of the diaphragm (the most sensitive); Diaphragmatic discontinuity; Diaphragmatic thickening sign; Collar sign; Herniation of abdominal viscera into the thorax; Dependent viscera sign	Pleural effusion (n = 8)/Lung contusion (n = 12)/Pneumothorax (n = 23)/Hemoperitoneum (n = 40)/Liver injury (n = 32)/Splenic injury (n = 16)/Kidney injury (n = 17)/Hollow viscus injury (33)/Fracture (n = 7)/Pneumoperitoneum (n = 4)	Sen: 94.44; Spe: 46.84; PPV: 44.74; NPV: 94.87
Leung, 2015	SW (70.59)/ GSW (29.41)	Focal diaphragmatic defects; Collar sign; Dependent viscera sign; Herniation of abdominal viscera or fat into the thorax; Hump sign; Band sign (The latter two signs were observed in right-sided TDI)	Hemothorax; Pneumothorax; Hemoperitoneum; Pneumoperitoneum (total n = 16)	Sen: 33.3; Spe: 100; PPV:100; NPV: 57.1
Melo, 2011	GSW	Focal thickening and focal discontinuity of the diaphragm; Pleural effusion (in all TDI cases)	Pleural effusion (in all TDI cases), ipsilateral (in 3 cases), asymmetrically bilateral (remaining), larger on the injured side	Sen: 87.5; Spe: 100; PPV: 100; NPV: 95.83
Stein, 2007	SW (63.76%) GSW (36.24%)	Discrete diaphragmatic disruption; Herniation of abdominal viscera or fat into the thorax; Contiguous injury sign on both sides of the diaphragm; Foreign body within the muscle of the diaphragm	NS	Sen: 76; Spe: 98.1; PPV: 95; NPV: 89.5; Accuracy: 90.9
Uhlich, 2018	NS	Herniation of abdominal viscera or into the thorax; Collar sign; Dependent viscera sign; Contiguous injury sign on both sides of the diaphragm; Diaphragmatic thickening sign; Curled diaphragm; Hump sign; Band sign; Discontinuous diaphragm; Dangling diaphragm	Hemothorax, traumatic brain injury, liver, renal, gastric, intestinal, pancreatic, and plenic injuries	Sen: 39.42; Spe: 96.65; PPV: 82; NPV: 80.5
Yucel, 2015	SW	Injury line crossing the diaphragm; Loss of diaphragmatic integrity; Diaphragmatic irregularity	NS	Sen: 82; Spe: 88; PPV:70.14; NPV: 93.43

*GSW* Gunshot wound, *MOI* Mechanism of injury, *NS* Not specified, *N* Number, *NPV* Negative predictive value, *PPV* Positive predictive value, *Sen* sensitivity, *Spe* specificity, *SW* stab wound, *TDI* Traumatic diaphragmatic injury

Among the included studies, the presence of a contiguous injury sign on both sides was found to be the most sensitive indicator of penetrating diaphragmatic injury. Common signs observed on MDCT scans indicating potential penetrating diaphragmatic injury included herniation of abdominal viscera or fat into the thorax, the collar sign, dependent viscera sign, transdiaphragmatic trajectory, diaphragmatic discontinuity, and diaphragmatic thickening. Additionally, associated injuries frequently observed with penetrating diaphragmatic injury included pleural effusion, pneumothorax, hemothorax, hemoperitoneum, and pneumoperitoneum (Table 3).

### Publication bias

Deek's funnel plot asymmetry test indicated no significant evidence of publication bias (P = 0.81) among the analyzed studies. As a result, we did not proceed with the trim-and-fill test (Supplementary Fig. 1).

### Quality assessment

Supplementary Fig. 2 provides a visual representation of the quality assessment of the included studies. Further details regarding the quality assessment for each individual study can be found in Supplementary Table 1. Overall, the majority of studies included in this review exhibited satisfactory methodological quality, indicating a low risk of bias and minimal concerns regarding applicability.

### Meta-analysis

**Fig. 2:**
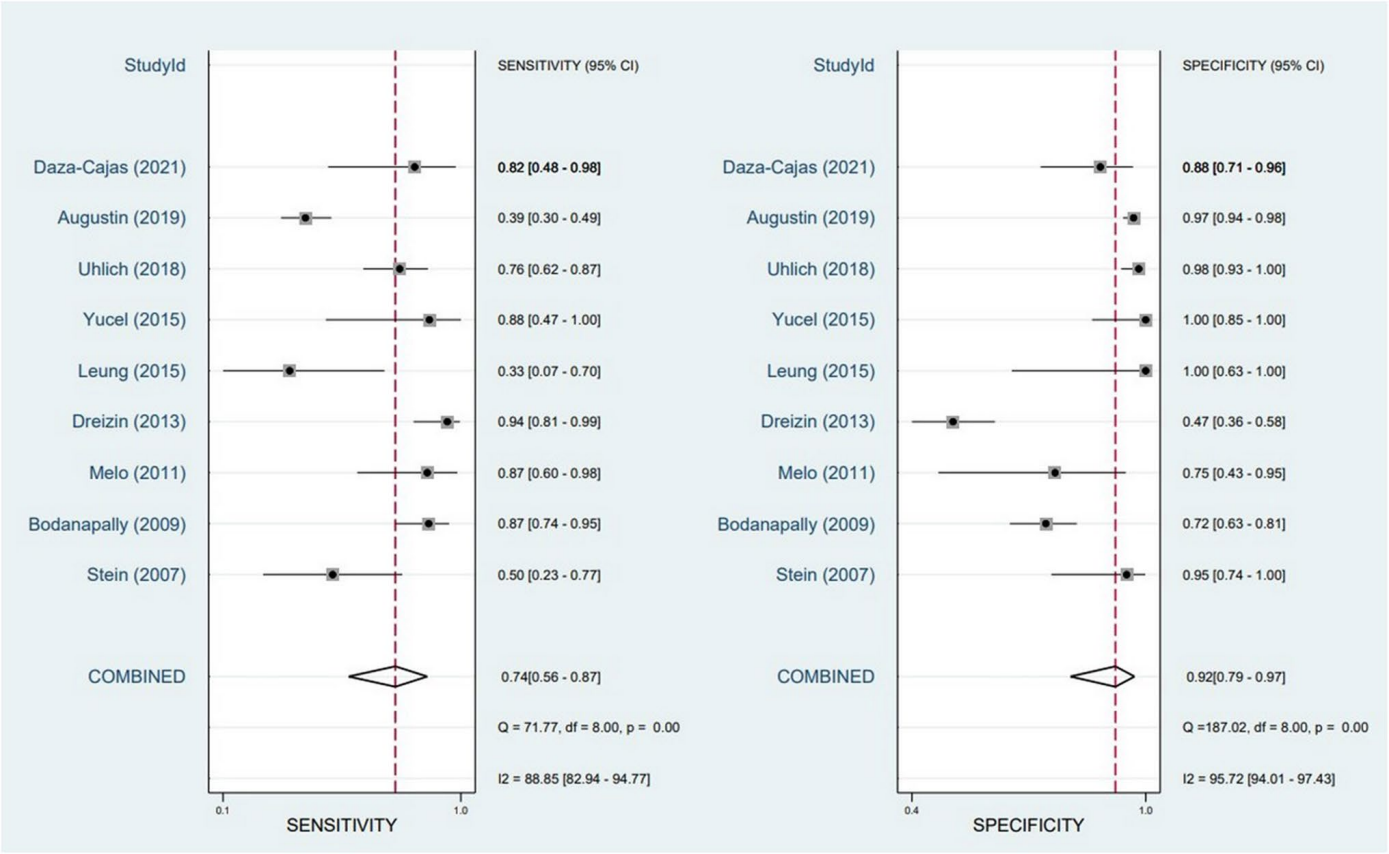
Forest plot and summary statistics of diagnostic test accuracy (DTA) meta-analysis of the included studies. CI: Confidence Interval

**Fig. 3:**
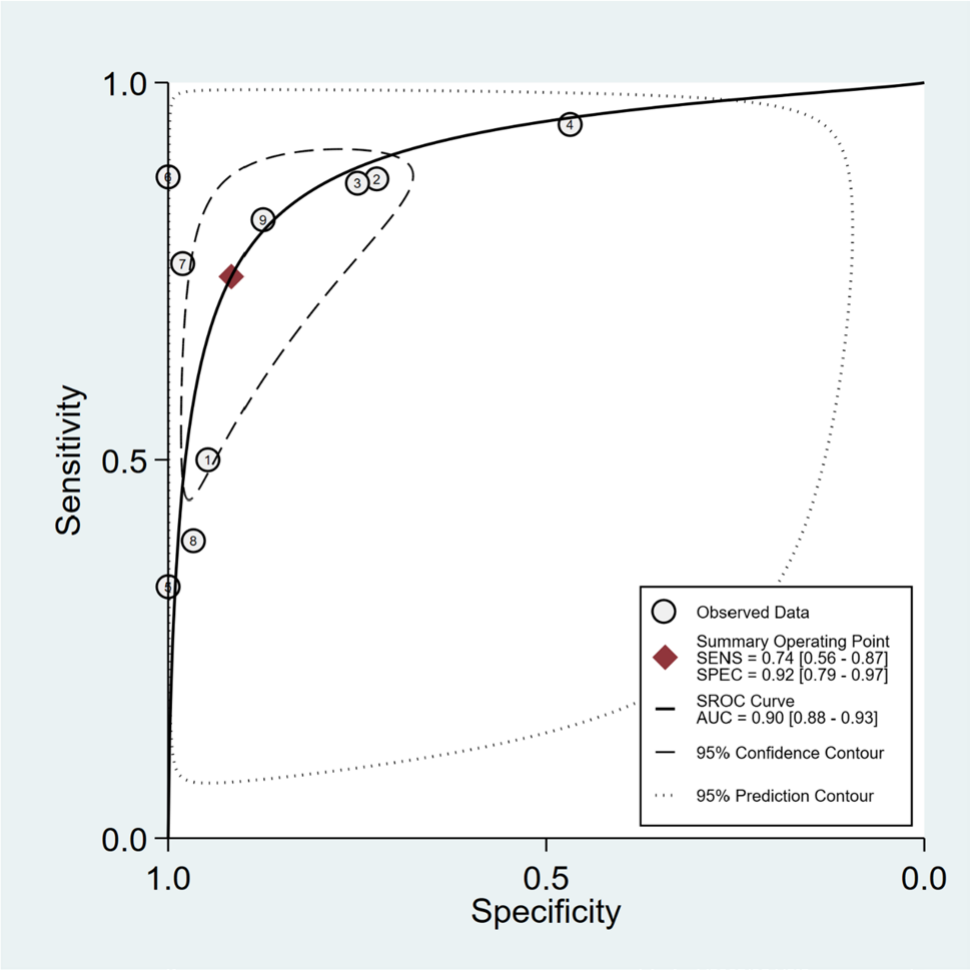
Summary receiver operating characteristic curve (SROC) of diagnostic test accuracy (DTA) meta-analysis of the included studies. AUC: Area Under the Curve. SENS: Sensitivity. SPEC: Specificity. SROC: Summary Receiver Operating Characteristic

The meta-analysis of nine studies investigating the diagnostic performance of MDCT in assessing diaphragmatic injury in penetrating trauma revealed a pooled sensitivity of 74% (95% CI: 56%-87%) and a pooled specificity of 92% (95% CI: 79%-97%) (Fig. 2). However, significant heterogeneity was observed in both sensitivity (I^2^ = 88.85%, 95% CI: 82.94–94.77) and specificity (I^2^ = 95.72%, 95% CI: 94.1–97.43) across the included studies. The SROC curve demonstrated an AUC of 0.90 (95% CI: 0.88–0.93) (Fig. 3).

We conducted univariate meta-regression analyses on several covariates, including mean age, mechanism of injury, and gender, to explore potential factors contributing to the heterogeneity observed. However, none of these covariates could account for the observed heterogeneity.

### Fagan plot analysis

**Fig. 4:**
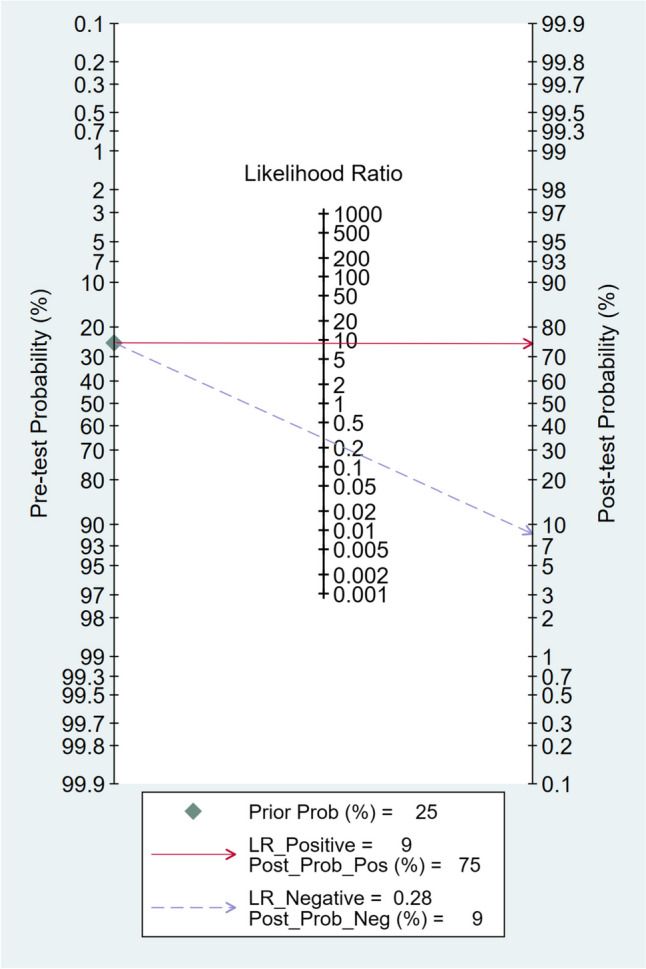
Fagan plot analysis using summary sensitivity and specificity results of the meta-analysis of the included studies with a pre-test probability of 25%

**Fig. 5:**
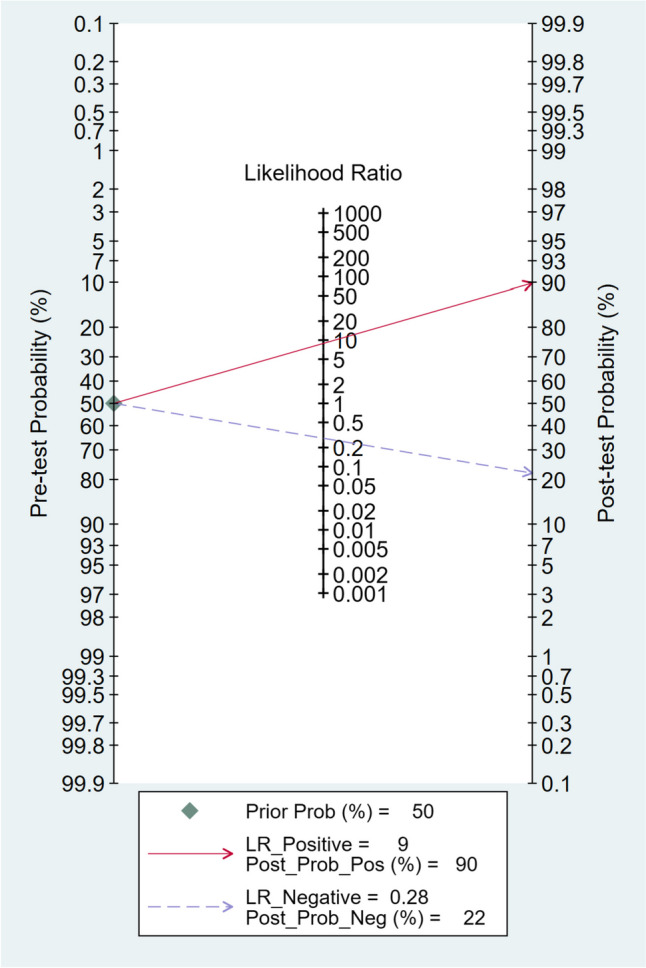
Fagan plot analysis using summary sensitivity and specificity results of the meta-analysis of the included studies with a pre-test probability of 50%

**Fig. 6:**
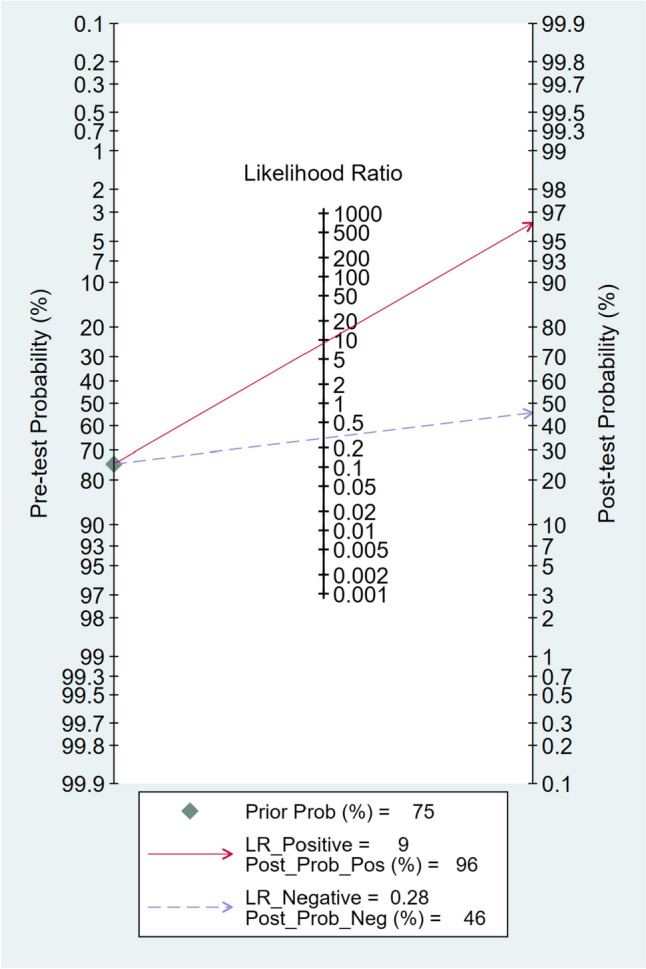
Fagan plot analysis using summary sensitivity and specificity results of the meta-analysis of the included studies with a pre-test probability of 75%

The Fagan plot analysis demonstrated that with pre-test probabilities of 25%, 50%, and 75%, the corresponding positive post-test probabilities were 75%, 90%, and 96%, respectively. Conversely, the negative post-test probabilities were 9%, 22%, and 46%, respectively. These findings are visually represented in Figs. 4, 5, and 6.

## Discussion

This systematic review and meta-analysis expand upon previous studies in examining the diagnostic effectiveness of MDCT in the detection of traumatic diaphragmatic injuries caused by penetrating trauma. The primary objective was to gather comprehensive data on different measures of diagnostic accuracy associated with MDCT as an imaging modality in this context.

Diaphragmatic injuries caused by penetrating thoracoabdominal trauma may initially be small and without symptoms [6, 22, 23]. However, if not diagnosed and treated promptly, these injuries can expand over time, leading to the herniation of abdominal organs into the chest cavity and the development of symptoms [5, 6]. Delayed treatment of such herniation is associated with increased complexity, mortality, and morbidity risks [5]. Injuries to the left hemidiaphragm are considered more significant because of the greater likelihood of organ herniation and strangulation, although they are relatively easier to detect compared to right-sided injuries that may be obscured by the liver [6]. The identification of diaphragmatic injuries can be challenging, particularly in cases of gunshot or stab wounds that cause small lacerations [6, 24]. However, techniques like identifying associated injuries or using tractography have shown promise in improving diagnostic accuracy [1, 7, 8].

In the management of diaphragmatic injury resulting from penetrating trauma, there has been a shift from routine laparotomy to a more conservative approach due to studies demonstrating that routine laparotomy was unnecessary and associated with significant mortality and morbidity [25–29]. This conservative therapy approach involves urgent laparotomy for unstable patients or those with signs of peritonitis, while a wait-and-see policy is applied to the remaining patients. Non-operative treatment is pursued for patients who do not require surgical therapy at the end of the conservative therapy period [5]. However, visualizing the diaphragm in asymptomatic patients who do not require surgery poses a challenge. Diagnostic laparoscopy has emerged as a preferred method, exhibiting high sensitivity and specificity [5, 24, 30]. However, it is important to note that routine laparoscopy is therapeutic in only one-third of cases, rendering it unnecessary in the remaining two-thirds [5]. According to a study by Kones et al., the rate of unnecessary diagnostic laparoscopies for penetrating injuries to the left thoraco-abdominal region was relatively high at approximately 56% [31]. These findings highlight the need for cautious evaluation before deciding to proceed with diagnostic laparoscopy. Consequently, various less invasive or non-invasive methods have been investigated as potential alternatives to laparoscopy. These methods include ultrasonography, peritoneal lavage, and chest X-ray. However, studies have shown that none of these methods were sufficiently sensitive to be implemented in clinical practice [5, 32–35]. Although some advocate for the use of magnetic resonance imaging (MRI) in diagnosing diaphragmatic injuries, this technique has not gained widespread use in the acute setting [2, 33].

Over the past decades, advancements in CT technology, particularly the introduction of MDCT, have enhanced the ability to detect and evaluate diaphragmatic injury resulting from penetrating trauma [8]. While CT has already established its value in assessing hemodynamically stable blunt abdominal trauma patients and has become the preferred imaging modality in this context, its application in penetrating thoracoabdominal trauma cases is still an area of active study [8, 36]. MDCT allows for rapid scanning of a large region of interest within a breath-holding interval, reducing imaging time and minimizing artifacts caused by respiratory movement [37]. This results in improved image quality and the ability to obtain thinner sections. Furthermore, the use of special digital software enables reconstruction of axial images into coronal, sagittal, and oblique planes, aiding in the identification of challenging anatomical structures or injuries [32, 33]. Various signs, such as herniation of abdominal organs, focal diaphragmatic defects, and discontinuity of the diaphragm, have been described to aid in the detection of penetrating diaphragmatic injury on MDCT scans [8]. Despite the difficulties associated with visualizing diaphragmatic injuries, MDCT offers superior sensitivity and specificity compared to conventional CT in identifying small, asymptomatic diaphragmatic injuries after penetrating thoracoabdominal wounds [32, 33].

In a meta-analysis focusing on blunt traumatic diaphragmatic injuries, Reitano et al. [34] demonstrated that contrast-enhanced computed tomography exhibited a notable level of sensitivity (80%) and specificity (98%) in the detection of such injuries. When it comes to diagnosing diaphragmatic injuries resulting from penetrating trauma, the reported sensitivity of MDCT displays considerable variability. This variability is illustrated by findings that range from as low as 33.3%, as observed in the study conducted by Lenung et al. [21], to as high as 94.44%, as reported in the investigation conducted by Daza-Cajas et al. [20]. Additionally, the specificity of this modality has been reported from 46.84% in Daza-Cajas et al.'s study [20] to 100% in the studies by Lenung et al. [21] and Melo et al. [8]. Our meta-analysis revealed a pooled sensitivity rate of 74% and specificity rate of 92% for MDCT in diagnosing penetrating diaphragmatic injury. However, significant heterogeneity was observed in both sensitivity and specificity among the included studies. Despite conducting univariate meta-regression analyses, none of the examined covariates could account for the observed heterogeneity. Due to limited data availability, it was not possible to include other covariates that could potentially address the heterogeneity. This variation in sensitivity and specificity across studies can be attributed to several other factors. Differences in study populations, including variations in the severity of penetrating trauma, as well as variances in methodology such as image acquisition protocols, interpretation criteria, type of CT scan utilized (e.g., thoracic, abdominal, thoracoabdominal), and characteristics of the MDCT device, can contribute to the observed variation in sensitivity and specificity. Factors such as the size and location of the injuries, as well as the level of expertise in interpreting MDCT images, may also impact the diagnostic performance. It is crucial to take into account the moderate to high level of diagnostic accuracy exhibited by MDCT in identifying diaphragmatic injury resulting from penetrating trauma, considering the specific patient population and clinical scenario. While MDCT demonstrates the ability to detect a significant proportion of penetrating diaphragmatic injuries and aid in ruling them out, there is still room for improvement. The observed heterogeneity, emphasize the need for further research and standardization in this area. Future studies should aim to clarify the factors contributing to the variability and work towards improving the consistency and reliability of MDCT in diagnosing penetrating diaphragmatic injuries.

Additionally, the Fagan plot analysis demonstrated that a higher pre-test probability was associated with a higher positive post-test probability, indicating a stronger association between a positive MDCT result and the presence of diaphragmatic injury. Conversely, when the MDCT scan produced a negative result, the probability of having diaphragmatic injury decreased. However, even with a negative result, there remained a small chance of having diaphragmatic injury, particularly in cases with higher pre-test probabilities. These findings acknowledge MDCT's value as a diagnostic tool for penetrating diaphragmatic injury. However, interpreting MDCT results necessitates taking into account the clinical presentation context and the initial likelihood of a fracture.

## Conclusion

This study emphasized the diagnostic effectiveness of MDCT in detecting diaphragmatic injury caused by penetrating trauma, especially in hemodynamically stable patients with no clear indications for immediate operative exploration. The meta-analysis revealed moderate to high diagnostic accuracy, with a pooled sensitivity of 74% and specificity of 92% for MDCT. However, further research with larger sample sizes, multicenter collaborations, and prospective designs is needed to investigate the factors contributing to the observed heterogeneity. This will help enhance our understanding and improve the consistency of MDCT in diagnosing diaphragmatic injury resulting from penetrating trauma.

### Supplementary Information

Below is the link to the electronic supplementary material.

Supplementary file1 (DOCX 128 KB)

## Data Availability

The datasets analyzed during the current study are available from the corresponding author on reasonable request.

## Declarations

### Leadership role

Ali Gholamrezanezhad serves as the Director of Clinical Research at the Department of Radiology in the Keck School of Medicine of USC.
