# Supplementary material for: Diagnostic utility of multidetector CT scan in penetrating diaphragmatic injuries: A systematic review and meta-analysis
Source: Emerg Radiol. 2023 Oct 4;30(6):765–76. doi: 10.1007/s10140-023-02174-1 (PMC10695863; doi:10.1007/s10140-023-02174-1)
Supplement: Supplementary file 1 — Supplementary file1 (DOCX 128 KB) [file 10140_2023_2174_MOESM1_ESM.docx]

| **Supplementary Table 1.** Methodological quality of the included studies according to the Quality Assessment of Diagnostic Accuracy Studies 2 checklist (QUADAS-2) | | | | | | | |
| --- | --- | --- | --- | --- | --- | --- | --- |
| **Study** | **RISK OF BIAS** | | | | **APPLICABILITY CONCERNS** | | |
|  | **PATIENT SELECTION** | **INDEX TEST** | **REFERENCE STANDARD** | **FLOW AND TIMING** | **PATIENT SELECTION** | **INDEX TEST** | **REFERENCE STANDARD** |
| Augustin, 2019 | ☺ | ☺ | ☺ | ☺ | ☺ | ☺ | ☺ |
| Bodanapally, 2009 | ☺ | ☺ | ☺ | ☹ | ☺ | ☹ | ☺ |
| Dreizin, 2013 | ☹ | ☺ | ☺ | ☹ | ☺ | ☺ | ☺ |
| Daza-Cajas, 2021 | ☺ | ☺ | ☺ | ☺ | ☹ | ☺ | ☺ |
| Leung, 2015 | ☹ | ☺ | ? | ☺ | ☺ | ☺ | ☺ |
| Melo, 2011 | ☺ | ☺ | ☺ | ☺ | ☹ | ☺ | ☺ |
| Stein, 2007 | ☺ | ☺ | ☺ | ☹ | ☺ | ☹ | ☺ |
| Uhlich, 2018 | ☺ | ☺ | ☺ | ☺ | ☺ | ☹ | ☺ |
| Yucel, 2015 | ☺ | ☺ | ☺ | ☹ | ☺ | ☺ | ☺ |
| ☺Low Risk; ☹High Risk; ? Unclear Risk | | | | | | | |


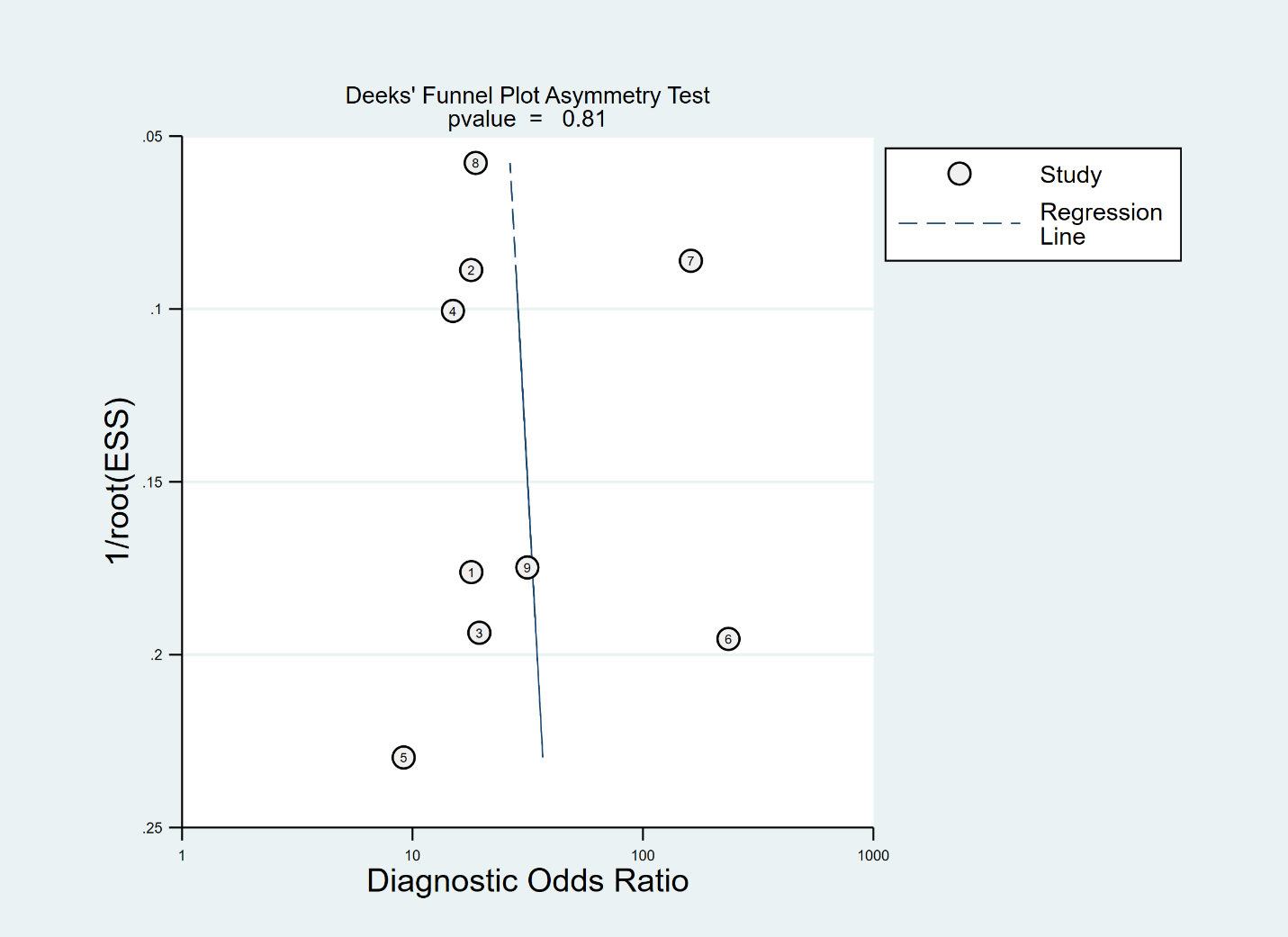


**Supplementary Fig. 1** Deek's funnel plot asymmetry test for publication bias assessment. (1) Augustin et al; (2) Bodanapally et al; (3) Dreizin et al; (4) Daza-Cajas et al; (5) Leung et al; (6) Melo et al; (7) Stein et al; (8) Uhlich et al; (9) Yucel et al


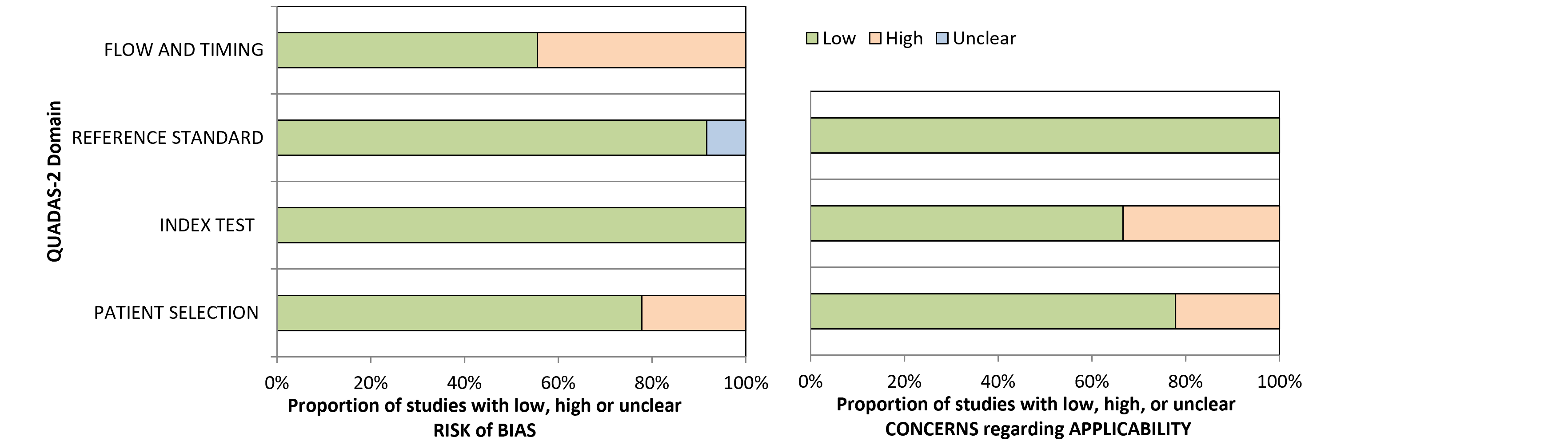


**Supplementary Fig. 2** Methodological quality assessment of the studies according to the Quality Assessment of Diagnostic Accuracy Studies 2 checklist (QUADAS-2)
